# Antimicrobial resistance among indicator *Enterococcus faecium* and *Escherichia coli* in Swedish pig farms

**DOI:** 10.1186/s13028-024-00756-8

**Published:** 2024-07-17

**Authors:** Valeriia Ladyhina, Susanna Sternberg-Lewerin, Linus Andersson, Elisabeth Rajala

**Affiliations:** 1 Division of Bacteriology and Food Safety, Department of Animal Biosciences, Swedish University of Agricultural Sciences, P.O. Box 7054, 750 07 Uppsala, Sweden; 2 Department of Medical Biochemistry and Microbiology (IMBIM), Uppsala University, P.O. Box 582, 751 23, Uppsala, Sweden

**Keywords:** AMR, Environmental sampling, Indicator bacteria, Pig, Surveillance, Swine

## Abstract

Monitoring the use of antimicrobials and the emergence of resistance in animals and people is important for the control of antimicrobial resistance, and for establishing sustainable and effective disease management practices. In this study, we used *Enterococcus* spp. and *Escherichia coli* as indicator species to investigate antimicrobial susceptibility patterns and how these change over time, on ten Swedish pig farms. Indoor environmental sock sampling was performed once a month during the entire production cycle of one batch of pigs on each farm, resulting in 60 samples collected in total. Selective culture for *E. coli* and *Enterococcus* spp. resulted in 122 isolates of *E. coli*, 74 isolates of *E. faecium*, but no isolates of *E. faecalis*. Microdilution was used to determine minimum inhibitory concentrations for twelve antimicrobial substances in *E. coli* and fifteen substances in *E. faecium*. The overall prevalence of resistance was low. Among the *E. coli* isolates, the proportions non-wild type (resistant, NWT) isolates were as follows: azithromycin and amikacin 1% (n = 1), trimethoprim and sulfamethoxazole 2% (n = 3), ampicillin 6% (n = 7) and tetracycline 9% (n = 11). Among the *E. faecium* isolates, the NWT proportions were: teicoplanin, linezolid and gentamicin 1% (n = 1), daptomycin 3% (n = 2), erythromycin 26% (n = 19), tetracycline 27% (n = 20), quinupristin/dalfopristin 58% (n = 42). The resistance patterns differed between the farms, likely due to different antimicrobial use, biosecurity measures and source of the animals. The NWT prevalence among *E. coli* decreased over time, whereas no similar trend could be observed in *E. faecium*. The results of the current study illustrate the complex factors affecting the antimicrobial resistance patterns observed on each farm, indicating that specific practices and risk factors have an impact on the prevalence and type of antimicrobial resistance. Further studies of the farm environments in combination with antimicrobial use and other risk factor data are needed to elucidate the multifaceted drivers of antimicrobial resistance development on livestock farms.

**Supplementary Information:**

The online version contains supplementary material available at 10.1186/s13028-024-00756-8.

## Findings

Antimicrobial resistance (AMR) is a serious health threat for animals and humans that requires urgent actions [1]. The consequences of AMR for animals are similar to those for humans e.g., treatment failures leading to suffering and decreased welfare, increased mortality, and reduced productivity with major impact on livelihoods and global food security [2]. Monitoring the use of antimicrobials and the emergence of resistance in animals and people is important for the control of AMR, and for establishing sustainable and effective disease management practices. The overall aim of this study was to investigate the prevalence of AMR in selected Swedish pig farms using two indicator bacteria, *Escherichia coli* and *Enterococcus* spp. Specific objectives were to (i) investigate if AMR differed between the farms, (ii) how AMR changed over time, and (iii) to assess the differences in the temporal dynamics of the resistance patterns between the two indicator species.

The material used in this study originated from environmental sock samples (boot swabs) obtained in 2023 from ten farrow-to-finish pig farms located at a maximum two hours driving distance from Uppsala. Sock samples have proven to be efficient for obtaining a representative picture of the bacteria that are present in a pig herd [3]. One group of pigs from each farm was selected for the 6 month study, with monthly visits throughout the entire production cycle. A total of 60 samples were collected, one pooled sample per herd and sampling occasion. The method is described here in brief, for more details see Additional file 1. For each sampling occasion, samples were kept cold and immediately transported to the laboratory at the Swedish University of Agricultural Sciences (SLU). Upon arrival at the laboratory, material was extracted from the sock samples by immersing them in sterile buffered peptone water. The samples were then processed in a stomacher, followed by centrifugation for sample concentration. Finally, they were preserved in 86% glycerol and stored at −80 °C for long-term storage. The thawed sample eluate was inoculated onto selective agar plates, MacConkey agar for detection of *E. coli*, and Slanetz and Bartley (SlaBa) for detection of *Enterococcus* spp. From each sample, two isolates with typical morphology were selected and Matrix Assisted Laser Desorption/Ionizaton–Time-of-Flight (MALDI-TOF) was used to confirm the identification of the isolates. Microdilution using Sensititre^™^ (ThermoFisher Scientific Inc., Waltham, MA, USA) panels was used to determine minimum inhibitory concentrations (MIC) for twelve antimicrobial substances in *E. coli* and fifteen substances in *Enterococcus* spp. Epidemiological cut-off values for the MIC, as determined by the European Committee on Antimicrobial Susceptibility Testing (EUCAST) [4] were used to classify isolates as belonging to the wild-type drug-susceptible population or the non-wild type (NWT) population and likely to be resistant to the tested drug. Data analysis and descriptive statistics were done in Microsoft^®^ Excel and data visualization was performed with R (v4.3.1) [5] using package ggplot2 (v3.4.4) [6].

**Fig. 1:**
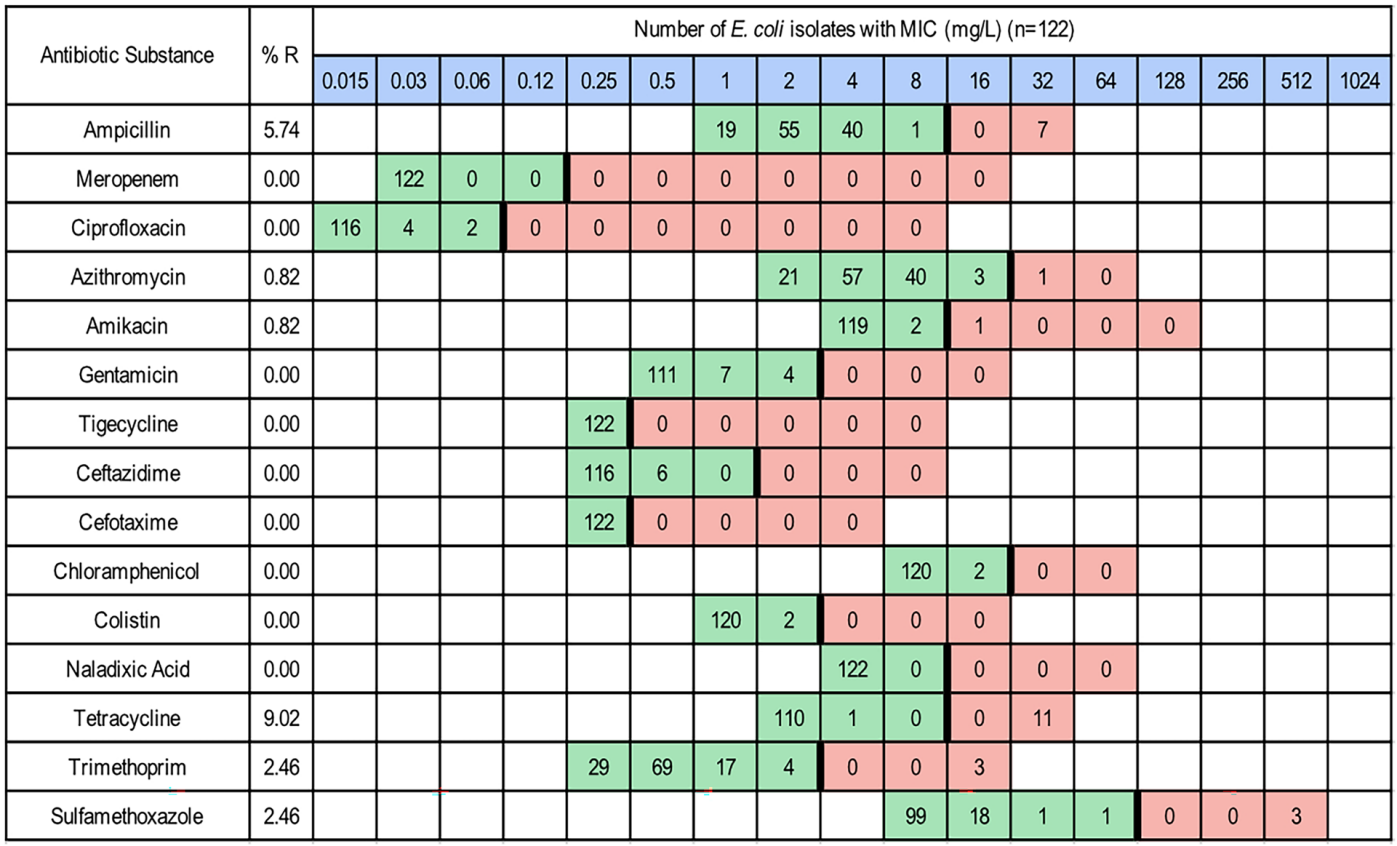
Distribution of MIC values of *E. coli* isolates (n = 122). Red and green cells indicate the range of tested concentrations. Vertical black lines indicate EUCAST epidemiological cutoffs

**Fig. 2:**
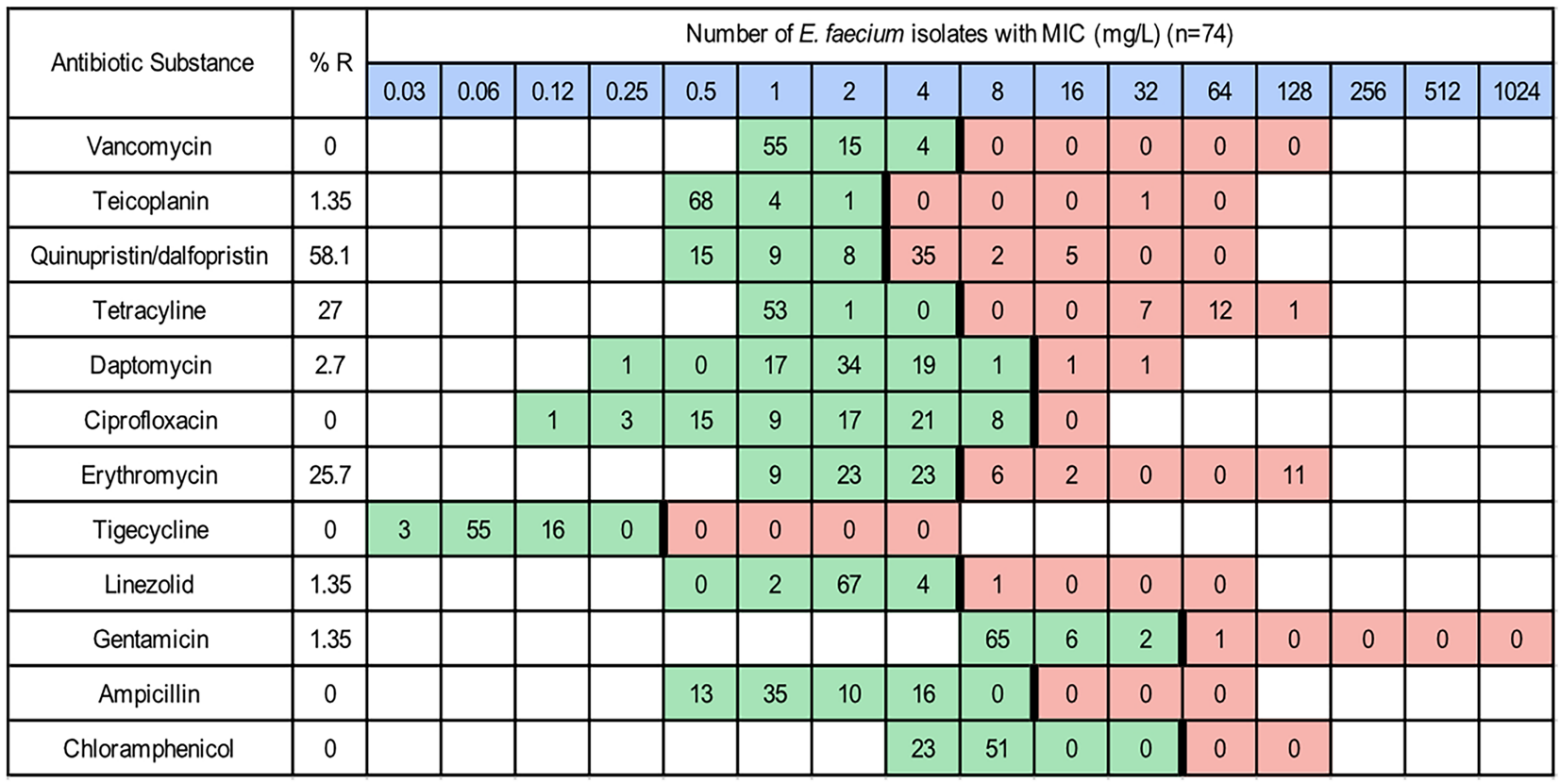
Distribution of MIC values of *E. faecium* isolates (n = 74). Red and green cells indicate the range of tested concentrations. Vertical black lines indicate EUCAST epidemiological cutoffs

**Fig. 3:**
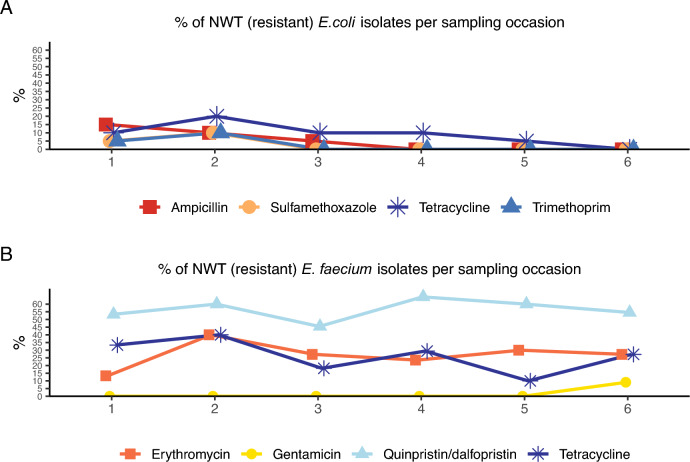
Proportions of non-wild type isolates from Swedish pig farms over a 6-month period. A. *E. coli* isolates (n = 122), B *E. faecium* (n = 74)

**Fig. 4:**
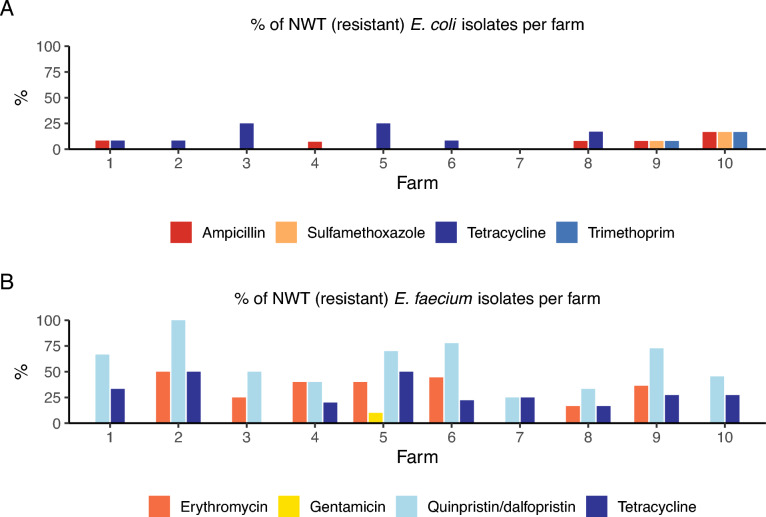
Proportions of non-wild type isolates from 10 Swedish pig farms. A. *E. coli* isolates (n = 122), B *E. faecium* (n = 74)

From the 60 samples, 122 isolates of *E. coli*, 74 isolates of *E. faecium*, but no isolates of *E. faecalis,* were identified and further analysed. The proportions of NWT (resistant) *E. coli* were as follows: azithromycin and amikacin 1% (n = 1), trimethoprim and sulfamethoxazole 2% (n = 3), ampicillin 6% (n = 7) and tetracycline 9% (n = 11) (Fig. 1). Among the *E. faecium* isolates, the NWT (resistant) proportions were: teicoplanin, linezolid and gentamicin 1% (n = 1), daptomycin 3% (n = 2), erythromycin 26% (n = 19), tetracycline 27% (n = 20), quinupristin/dalfopristin 58% (n = 42) (Fig. 2). A majority of the *E. faecium* isolates classified as NWT (resistant) to quinupristin/dalfopristin, tetracycline and erythromycin had MIC values just above the epidemiological cutoff. Among the *E. coli* isolates, AMR decreased over time (Fig. 3A), while no such trend could be observed in the *E. faecium* isolates (Fig. 3B). The AMR patterns for each farm were different for both bacteria (Fig. 4). All farms except farm 7 had *E. coli* isolates that exhibited resistance to either ampicillin or tetracycline, or both, at some point during the production cycle (Fig. 4A). From farms 7, 8, 9 and 10, there were also isolates that were NWT (resistant) to at least one of the following substances: azithromycin, amikacin, trimethoprim or sulfamethoxazole. All farms yielded *E. faecium* isolates NWT (resistant) to quinupristin/dalfopristin. In addition, all farms except farm 3 had isolates with resistance to tetracycline at some point in the production cycle (Fig. 4B).

The results demonstrated differences in AMR patterns between the investigated pig farms, likely due to variation in antimicrobial use and other risk factors between farms, as previously reported [7]. The overall prevalence of AMR in *E. coli* was low, which is in line with previous published research in Sweden [7, 8]. A study from New Zealand presented similar findings as the current study with *E. coli* being susceptible for ciprofloxacin, but NWT (resistant) for ampicillin and tetracycline [9]. In contrast, a study in Spanish pig herds demonstrated much higher proportions of NWT *E. coli*, for most antibiotics tested [10]. The overall prevalence of AMR in *E. faecium* was higher compared to *E. coli,* this was also similar to the New Zealand study [9]. The higher AMR prevalence in *E. faecium* could partly be explained by this species exhibiting intrinsic low susceptibility to certain antimicrobials, e.g., quinupristin [11]. All *E. faecium* isolates in the current study were susceptible to ampicillin, and vancomycin, while resistance to erythromycin was seen on nearly all farms. Similar prevalence of resistance to erythromycin and tetracycline in *E. faecium* have previously been reported in Estonia [12], but higher prevalence has been shown in southern Europe (Portugal [13] and Spain [10, 14]). The current study also showed that AMR in *E. coli* decreased during the lifespan of the pigs, which is in line with previous research [15, 16]. This result reflects common practices of antimicrobial use, where most treatments in pig production in Sweden are administered to piglets [7].

The initial plan was to isolate both *E. faecium* and *E. faecalis*. However, most samples yielded no colonies of *E. faecalis*, despite a repeated attempt to isolate these from the stored frozen samples. This may be due to the sampling methodology, while individual fecal sampling or rectal swabs might have made detection of *E. faecalis* isolates more feasible. However, other European studies have also shown a higher prevalence of *E. faecium* than *E. faecalis* in faecal samples from pig farms [10, 16, 17]. The predominant isolation of *E. faecium* can be explained by its presence in typical fecal microbiota and its tendency to survive longer than other enterococci on dry material [18]. In addition, the microdilution method, with subsequent two-fold dilution steps may yield a one-step deviation in MIC results [4]. This is particularly of note when considering results that are just above or below cutoff, such as the quinupristin/dalfopristin resistance observed in our *E. faecium* isolates. However, this would only have had a small effect on the results of this study, as most MIC results leading to classification of an isolate as NWT (resistant) were above the cutoff by more than one step.

In conclusion, the overall prevalence of AMR in *E. coli* was low among the tested pig herds, while a higher prevalence of AMR was observed in *E. faecium*. Furthermore, the AMR prevalence differed between farms and decreased with age among *E. coli*. The results of the current study emphasize the complex factors leading to the specific AMR pattern observed on a specific farm. Further studies of entire farm environments in combination with data on antimicrobial use and other risk factors are needed to elucidate the multifaceted drivers of AMR development.

### Supplementary Information

Supplementary Material 1.

## Data Availability

All data are available from the corresponding author upon reasonable request.
