# Correction to: Antimicrobial resistance among indicator Enterococcus faecium and Escherichia coli in Swedish pig farms

**DOI:** 10.1186/s13028-024-00758-6

**Published:** 2024-08-13

**Authors:** Valeriia Ladyhina, Susanna Sternberg‑Lewerin, Linus Andersson, Elisabeth Rajala

**Affiliations:** 1 Division of Bacteriology and Food Safety, Department of Animal Biosciences, Swedish University of Agricultural Sciences, P.O. Box 7054, Uppsala, 750 07 Sweden; 2 Department of Medical Biochemistry and Microbiology (IMBIM), Uppsala University, P.O. Box 582, Uppsala, 751 23 Sweden

**Correction to: Antimicrobial resistance among indicator Enterococcus faecium and Escherichia coli in Swedish pig farms**

10.1186/s13028-024-00756-8

Following publication of the original article [1], we have been notified that Figs. 1 and 2 were published incorrectly.

**Fig. 1:**
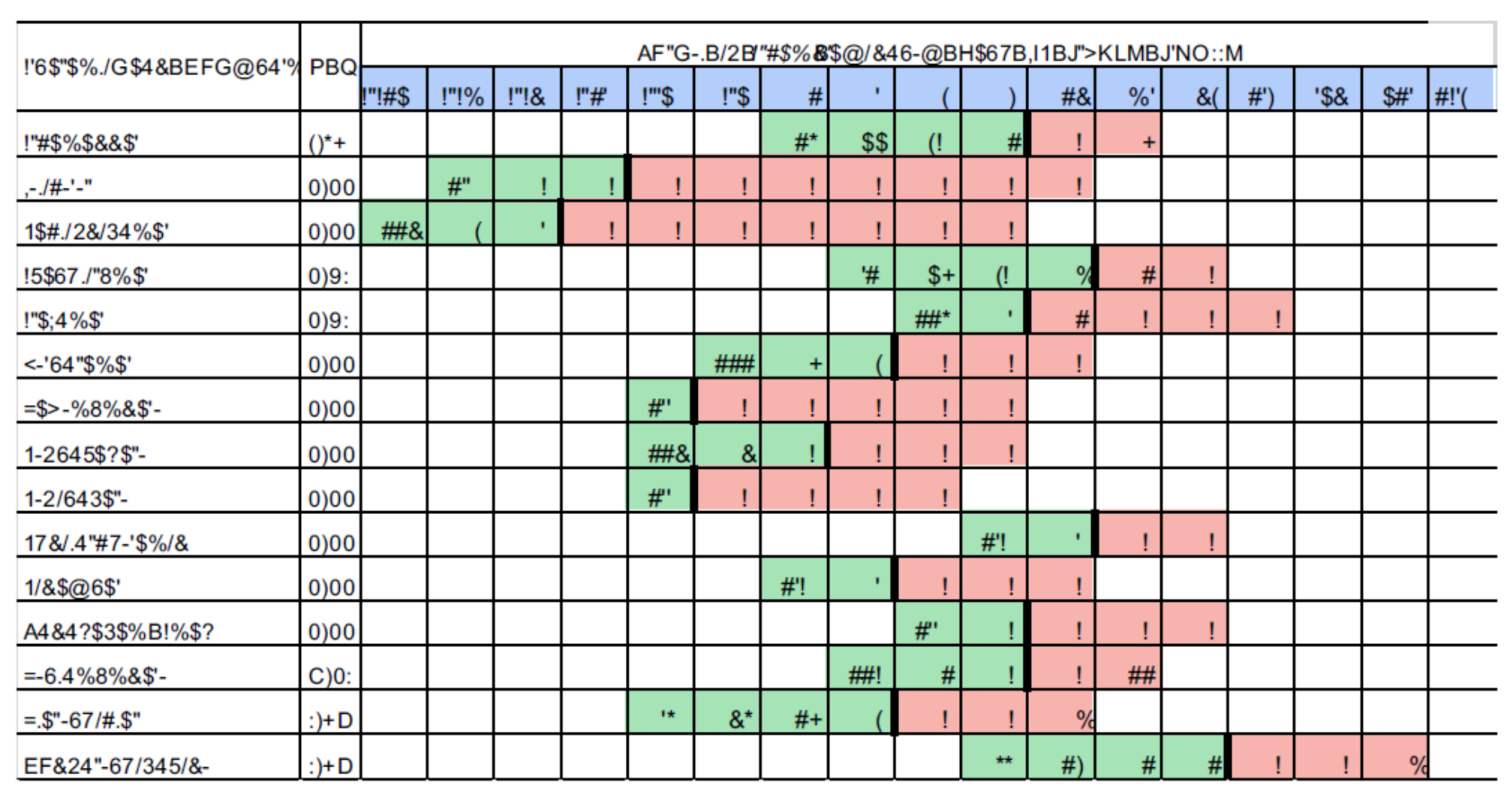
Distribution of MIC values of *E. coli* isolates (*n* = 122). Red and green cells indicate the range of tested concentrations. Vertical black lines indicate EUCAST epidemiological cutoffs

**Fig. 2:**
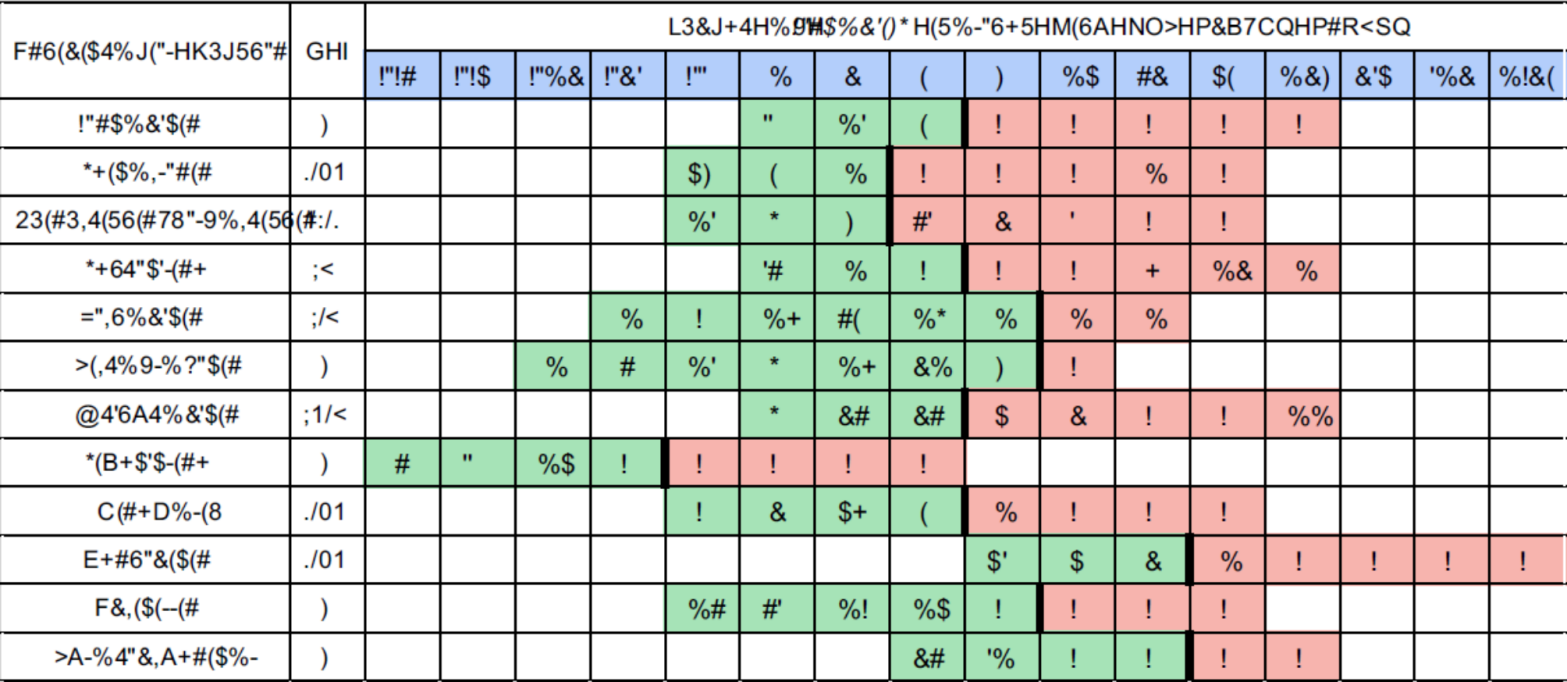
Distribution of MIC values of *E. faecium* isolates (*n* = 74). Red and green cells indicate the range of tested concentrations. Vertical black lines indicate EUCAST epidemiological cutoffs

It should be as follows:

**Fig. 1:**
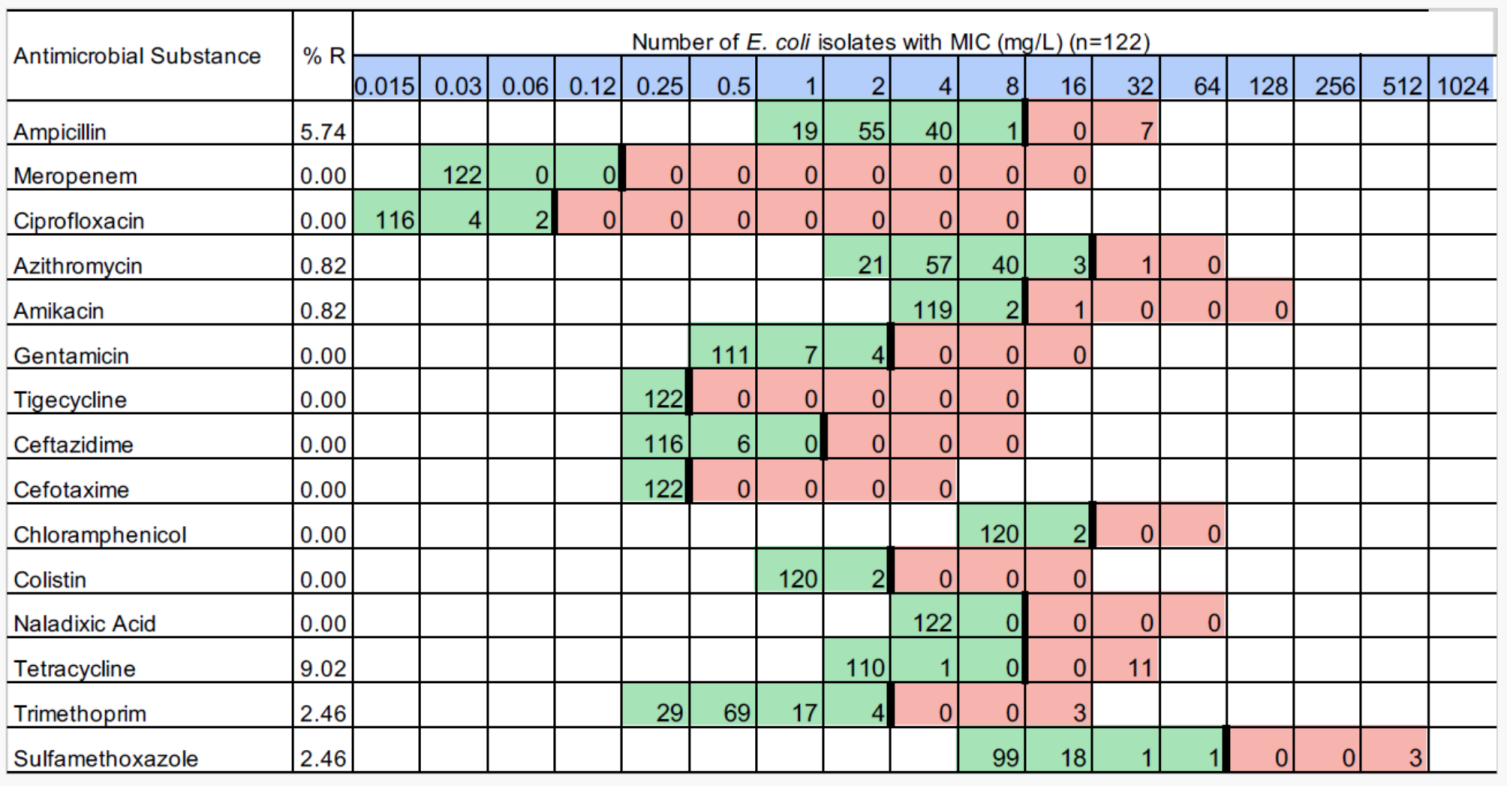
Distribution of MIC values of *E. coli* isolates (*n* = 122). Red and green cells indicate the range of tested concentrations. Vertical black lines indicate EUCAST epidemiological cutoffs

**Fig. 2:**
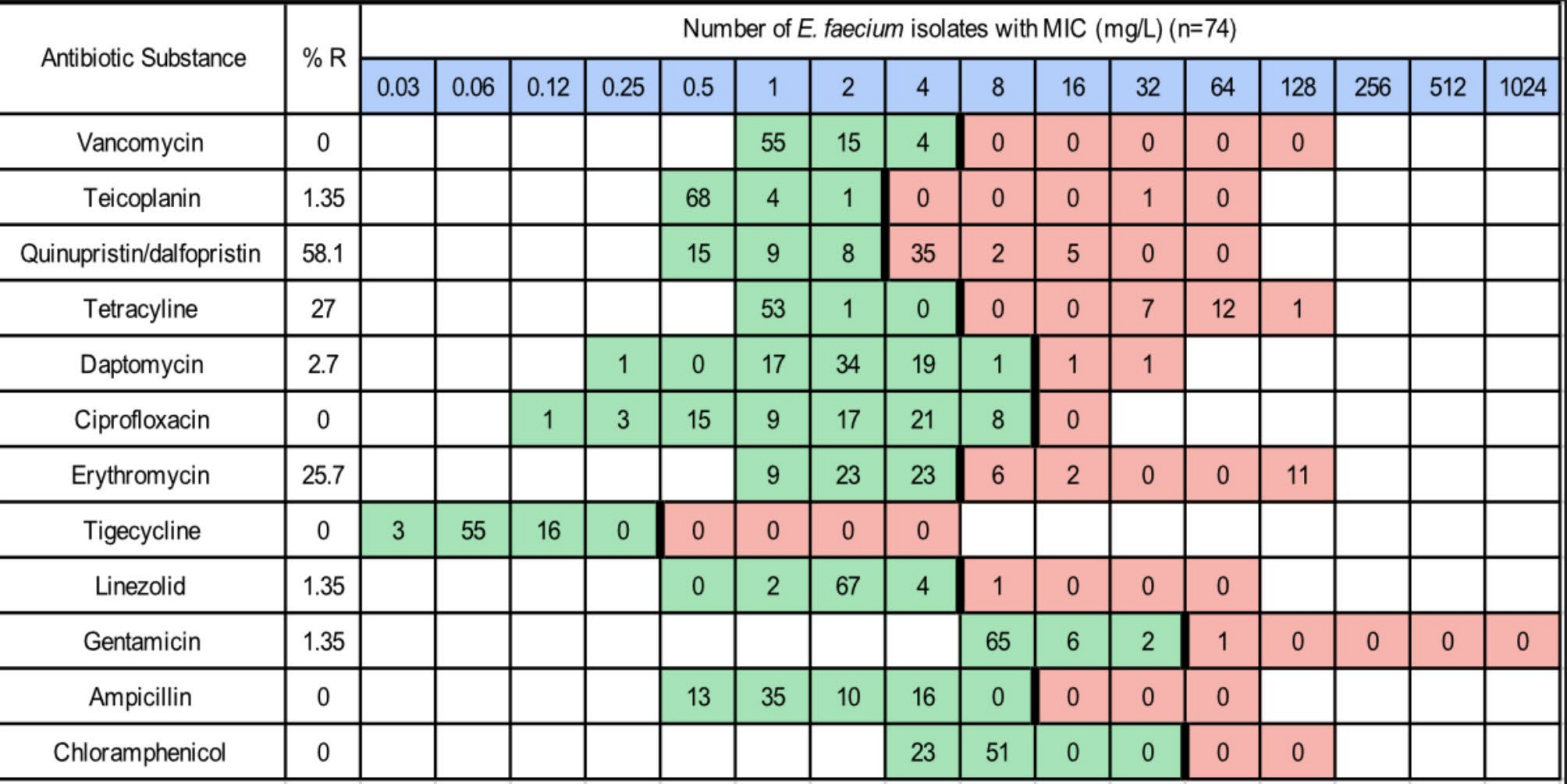
Distribution of MIC values of *E. faecium* isolates (*n* = 74). Red and green cells indicate the range of tested concentrations. Vertical black lines indicate EUCAST epidemiological cutoffs

The original article was updated.

## References

[1] Ladyhina, et al. Antimicrobial resistance among indicator Enterococcus faecium and Escherichia coli in Swedish pig farms (2024). 66. 2024;34. 10.1186/s13028-024-00756-8. 10.1186/s13028-024-00756-8 PMC11256653 39020377

