# Supplementary material for: Antimicrobial resistance among indicator Enterococcus faecium and Escherichia coli in Swedish pig farms
Source: Acta Vet Scand. 2024 Jul 17;66:34. doi: 10.1186/s13028-024-00756-8 (PMC11256653; doi:10.1186/s13028-024-00756-8)
Supplement: Supplementary file 1 — Supplementary Material 1. [file 13028_2024_756_MOESM1_ESM.pdf]

Methods

The material used in this study originated from environmental sock samples (boot swabs) obtained from ten Swedish farrow-to-finish pig farms. One batch of pigs on each farm was followed by monthly sampling visits during the 6-month production cycle. The sampling was performed from February 2023 until August 2023 and started when the piglets reached an age of ~1 week and ended 1-2 weeks before slaughter. Depending on the number of pens, 3 or 4 pairs of socks samples were collected per farm visit. One hundred steps were taken in each pair of socks and sampling was performed in such way as to equally cover the whole group of pigs spatially. For most of the farms, collection of samples was performed in different pens in the same building. However, starting from the age of 2 months in farm 5 and 6, the pig group of interest was distributed in 2 different buildings. To avoid contamination from the environment around the pens of these pigs, additional plastic boot covers were used.

The samples were kept cold during transport to the laboratory at SLU immediately after sampling. Upon arrival at the lab, 50 ml of sterile buffered peptone water (BPW) was added to each sample and extraction performed by stomacher. After centrifugation at 3000g for ten minutes, the pellet was resuspended in 15 ml of BPW. All samples from each visit were pooled into one, resulting in a total of 60 samples (one pooled sample per farm per visit). In order to preserve the bacteria, glycerol 86 % was added to the samples before storage at -80°C.

For this study, the samples were thawed, re-suspended in BPW to obtain a concentration of 0.4g/ml and vortexed before inoculating onto selective agar plates. For culture of *Escherichia coli*, MacConkey agar plates (one per sample) were incubated for 24 hours at 35°C, and for *Enterococcus* spp. Slanetz and Bartley agar plates (four per sample) were incubated for 48 hours at 44°C. Two typical colonies were picked from each plate and sub-cultured to obtain

pure cultures. Bacterial species confirmation was performed by Matrix Assisted Laser Desorption/Ionizaton–Time-of-Flight (MALDI-TOF).

Each confirmed isolate was subjected to antimicrobial susceptibility testing by Sensititre™ (ThermoFisher Scientific Inc., Waltham, MA, USA) panels, according to the manufacturer's instructions. Sensititre EUVENC panels were used for *Enterococcus faecium* and Sensititre EUVSEC3 were used for *E. coli*. Two control strains: *E. coli* (ATCC25922) and *Enterococcus faecalis* (ATCC29212), provided by the manufacturer, were used for quality control.

Epidemiological cut-off values, as determined by the European Committee on Antimicrobial Susceptibility Testing (EUCAST) were used to classify isolates as either wild-type drug-susceptible or non-wild type that likely to be resistant to the tested drug. Data collection and analysis was done in Microsoft® Excel (Microsoft Co. Redmond, WA, USA). Data visualisation was performed using Microsoft® Excel (Microsoft Co. Redmond, WA, USA), R (v4.3.1) and Rstudio (v2023.09.0+463). The majority of plots were visualized using the ggplot2 (v3.4.4) package.
